# Increase in cartilage degeneration in all knee compartments after failed ACL reconstruction at 4 years of follow-up

**DOI:** 10.1186/s10195-021-00618-3

**Published:** 2021-12-16

**Authors:** Kathleen Andrä, Robert Prill, Enes Kayaalp, Lars Irlenbusch, Eckehard Liesaus, Tilo Trommer, Peter Ullmann, Roland Becker

**Affiliations:** 1 Center of Orthopaedics and Traumatology, University of Brandenburg, Medical School “Theodor Fontane”, Hochstrasse 29, 14770 Brandenburg an der Havel, Germany; 2 SportsClinic Erfurt, Erfurt, Germany; 3 Department of Orthopedics and Traumatology, Istanbul Taksim Training and Research Hospital, Siraselviler Cad, Beyoglu, Istanbul, 34433 Turkey

**Keywords:** Anterior cruciate ligament reconstruction, ACL, Revision, Failure, Tunnel placement, Meniscal lesion, Cartilage degeneration

## Abstract

**Purpose:**

Degeneration of the cartilage after anterior cruciate ligament reconstruction (ACL-R) is known, and further deterioration can be expected in patients with tunnel malplacement or partial meniscal resection. It was hypothesized that there is a significant increase in cartilage degeneration after failed ACL-R.

**Material and methods:**

Isolated ACL revision surgery was performed in 154 patients at an interval of 46 ± 33 months (5–175 months) between primary and revision surgery. Cartilage status at the medial, lateral femorotibial, and patellofemoral compartments were assessed arthroscopically during primary and revision ACL-R in accordance with the Outerbridge classification. Tunnel placement, roof angle, and tibial slope was measured using anteroposterior and lateral radiographic views.

**Results:**

Cartilage degeneration increased significantly in the medial femorotibial compartment, followed by the lateral and patellofemoral compartments. There was a correlation between both cartilage degeneration in the patellofemoral compartment (PFC) (*r*_*s*_ = 0.28, *p* = 0.0012) and medial tibial plateau (*R*_*s*_ = 0.24, *p* = 0.003) in relation to the position of tibial tunnel in the frontal plane. Worsening of the cartilage status in the medial femorotibial compartment, either femoral or tibial, was correlated with the tibial aperture site in the lateral view (*R*_*s*_ = 0.28, *p* < 0.001). Cartilage degeneration in the lateral compartment of the knee, on both femoral or tibial side, was inversely correlated with the femoral roof angle (*R*_*s*_ = −0.1985, *p* = 0.02). Meniscal tears, either at the medial or lateral site or at both, were found in 93 patients (60%) during primary ACL-R and increased to 132 patients (86%) during revision ACL-R.

**Discussion:**

Accelerated cartilage degeneration and high prevalence of meniscal lesions are seen in failed ACL-R. Tunnel placement showed significant impact on cartilage degeneration and may partially explain the increased risk of an inferior outcome when revision surgery is required after failed primary ACL-R.

*Level of evidence:* Level IV—retrospective cohort study.

## Introduction

Cartilage degeneration at different sites of the knee occurs after anterior cruciate ligament reconstruction (ACL-R) [9, 14, 23]. Older age, higher body mass index (BMI), meniscal tears, and technical errors may accelerate cartilage degeneration after primary ACL-R [11, 19].

An evaluation of cartilage status after ACL-R with second-look arthroscopy indicated worsening of the cartilage status in all knee compartments, except for the lateral femoral condyle [4]. Interestingly, anterior laxity and meniscal lesions did not correlate with the deterioration of the cartilage status. However, degenerative change of normal cartilage was found in 45% of the entire study cohort [4].

In contrast to these findings after primary ACL-R, the current study focused on a special group of patients who had received a second arthroscopy due to failed ACL-R. Up to 15% of patients experience a re-tear or insufficiency after ACL-R [10]. Failure is mainly caused by another trauma, surgical errors during primary ACL-R, or biological factors. Nonanatomical tunnel placement causes revision of failed ACL-R in 22–79% of the cases [7, 11, 12, 29, 33]. The impact of anatomic and nonanatomic ACL-R on osteoarthritis was analyzed in a literature review, and an increased prevalence was found between 23.2% and 43.9% when tunnel placement was nonanatomical after 10 years of follow-up [28]. Significant acceleration of cartilage degeneration after failed ACL-R has been reported [2, 22, 24].

However, the site of the most pronounced cartilage degeneration in the knee remains unclear. Most studies used magnet resonance imaging (MRI) for cartilage assessment. MRI commonly underestimates cartilage damage when compared with arthroscopic assessment, emphasizing the relevance of the current study [15, 37]. No clear conclusions can be drawn with respect to the degrees and sites of cartilage degeneration from the currently available literature.

It was hypothesized that deterioration of the cartilage will occur after failed ACL-R and will be accelerated by both, tunnel malplacement and partial meniscal resection.

## Material and methods

Records and radiographies of 154 consecutive patients operated at the sport clinic, Erfurt, between January 2010 and December 2015 were evaluated in the retrospectively designed study. Meniscus lesion and cartilage degeneration at the medial, lateral femorotibial, and patellofemoral compartments were assessed during primary and revision ACL-R, in accordance with the Outerbridge classification [32].

The femoral and tibial tunnel position was measured using the anteroposterior and lateral view of radiographies after primary ACL-R. The angulation between both the tibial and femoral tunnel, and the intraarticular aperture site was measured in the coronal plane. The posterior slope of the tibial plateau, the femoral roof angle, the intraarticular aperture site of femoral and tibial bone tunnel, and the angle of the tibial bone tunnel to the joint line was measured in the sagittal plane (Table 1) [20].

**Table 1:** Definition of the radiographic measurements using the anteroposterior and lateral view

Tibial slope	Angle between the tibial plateau and the mechanical axis in the lateral view
Femoral roof angle	Angle between the Blumensaat’s line and the posterior cortical bone of the femur
Intraarticular aperture site of the tibial tunnel in the AP view	Aperture site in % = 100% × A/A + B A = distance from the medial border of the tibial plateau to the center of the tunnel B = distance from the lateral border of the tibial plateau to the center of the tunnel
Intraarticular aperture site of the tibial tunnel in the lateral view	Aperture site in % = 100% × A/A + B A = distance from the anterior border of the tibial plateau to the center of the tunnel B = distance from the posterior border of the tibial plateau to the center of the tunnel
Angle of the tibial tunnel in the AP view	Angle between the tibial plateau and the tibial tunnel
Angle of the tibial tunnel in the lateral view	Angle between the tibial plateau and the tibial tunnel
Intraarticular aperture of the femoral tunnel in the lateral view	Aperture site at the Blumsaat’s line in % = 100% × A/A + B A = distance from the posterior femoral cortical bone to the center of the tunnel B = distance from the anterior femoral condyle to the center of the tunnel
Femoral angle in the AP view	Angle between a line drawn perpendicular to the tibial plateau and the femoral tunnel
Graft angulation in the AP view	Angle between the graft orientation and the tibial plateau

Additionally, the cause of secondary instability was analyzed. According to the history of failure, the study cohort was divided into a trauma and nontrauma group. A factor analysis was performed between the two groups to study the impact of tunnel position on failure of ACL-R. Patients in the trauma group had experienced an adequate trauma during sports activity such as football, handball, wrestling, jogging, or alpine skiing. A lack of appropriate trauma was allocated to the nontrauma group.

### Statistical analysis

Descriptive statistics were used to present demographic data. Data were presented as mean ± standard deviation and range where appropriate. All variables were analyzed for normal distribution using the Shapiro–Wilkinson test. Nominal values between groups were correlated using the chi-square test. Independent group variables were analyzed by an unpaired *t*-test or Mann–Whitney *U*-test, depending on the distribution of normality.

Wilcoxon signed rank test was used to identify any significant difference between cartilage status, as classified by the Outerbridge classification, at two different time points: primary and revision ACL-R at five different locations of the knee such as (1) patellofemoral compartment, (2) medial femoral condyle, (3) lateral femoral condyle, (4) medial tibial plateau, and (5) lateral tibial plateau.

Difference in cartilage status, as defined by the Outerbridge classification, was calculated by subtracting the former cartilage grade (at primary ACL-R) from the latest cartilage grade (at revision ACL-R). The results were analyzed by the Spearman correlation test with the individual numeric radiographic measurements. A *p*-value of < 0.05 was regarded as statistically significant. All statistical analyses were performed using SPSS^©^ Statistics Version 27 (IBM Corporation, Armonk, NY, USA).

Ethical approval was granted by the ethical committee of the University at Jena (5115-03/17) and written consent was given by all patients.

## Results

Overall, 31 female and 123 male patients with a mean age of 23.9 ± 7.5 years (range, 11–57 years) at the time of primary ACL-R were included in the analysis. The right knee was affected in 52% of the included patients. All patients had undergone primary ACL-R using a quadrupled autologous hamstring graft (semitendinosus and gracilis tendons). Isolated ACL revision surgery was performed in 154 patients after a mean time of 46 ± 33 months (5–175 months).

The mean diameter of the quadrupled hamstring graft was 8 ± 1 mm in primary ACL-R. Transtibial drilling for the femoral tunnel was performed in 132 patients (85.7%) and anteromedial portal drilling in 22 patients (14.3%). Femorally, button fixation (Endobutton®, Smith & Nephew; Andover, MA, USA, ACL Tight rope, Arthrex Naples FL, USA) was performed in 118 patients, Rigidfix (Mitek-DePuySynthes Raynham, MA, USA) in 26 patients, and interference screw fixation (Fa. Storz, Tuttlingen, Germany) in 10 patients. Fixation on the tibial side was performed using suture disc (Fa. Storz, Tuttlingen, Germany) in 105 patients and an interference screw (Fa. Storz, Tuttlingen, Germany) in 49 patients.

**Fig 1:**
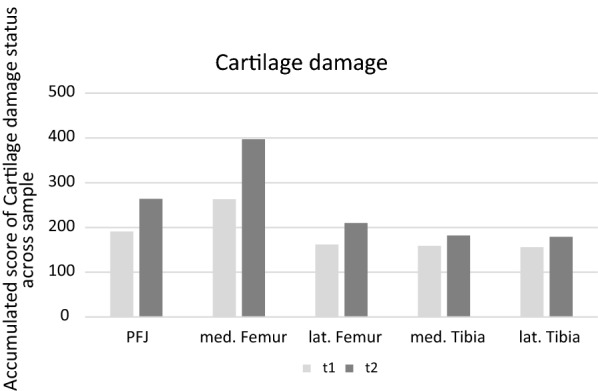
Accumulation of cartilage degeneration for all three compartments at t1 (primary ACL reconstruction) and t2 (revision ACL reconstruction)

There was a significant difference in cartilage status between primary and revision surgery in all knee compartments (Fig. 1). Increased cartilage degeneration was observed in comparison to the primary ACL-R in the patellofemoral compartment (*p* < 0.001), medial femoral condyle (*p* < 0.001*),* lateral femoral condyle (p < 0.001), medial tibial plateau (*p* = 0.01), and lateral tibial plateau (*p* = 0.003) (Table 2).

**Table 2:** Frequency of cartilage damage in number of patients at the time of primary and revision ACL surgery

Compartment	Patellofemoral		Medial tibia		Lateral tibia		Medial femur		Lateral femur	
ACL surgery	Primary	Revision	Primary	Revision	Primary	Revision	Primary	Revision	Primary	Revision
Grade 0	138	112	152	144	153	142	108	64	150	133
Grade 1	0	3	0	0	0	2	1	1	0	0
Grade 2	11	14	1	3	1	7	28	41	4	11
Grade 3	5	21	1	6	0	3	16	32	0	6
Grade 4	0	4	0	1	0	0	1	16	0	4

Data on the tibial slope, femoral roof angle, and the position of the tibial and femoral bone tunnel are summarized in Table 3.

**Table 3:** Measurements on the anteroposterior and lateral radiographic view

Measurements sagittal plane	Mean and SD
Posterior slope of the tibial plateau	6.8 ± 3.3°
Femoral roof angle	33.7 ± 4.6°
Aperture site of the tibial tunnel	40.4 ± 5.2%
Aperture site of the femoral tunnel on the Blumensaat line referenced to the posterior cortical bone	26.2 ± 7.3%
Angle of the tibial tunnel	62.4 ± 6.3°

**Measurements coronal plane**	**Mean and SD**
Intraarticular aperture site of the tibial tunnel	42.4 ± 3.2%
Angulation of the femoral tunnel	75.5 ± 4.0°
Angulation of the tibial tunnel	62.7 ± 7.2°

The aperture of the tibial tunnel averaged 42.1 ± 3.2% in the coronal plane and 40.4 ± 5.2% in the sagittal plane, relative to the size of the plateau.

Aperture site of the femoral tunnel was 26.2 ± 7.3% of the entire diameter of the lateral condyle in the lateral radiographic view. When the entire lateral femoral condyle was divided into four quarters from posterior to anterior parallel to the Blumensaat’s line, femoral aperture was present in the first quarter in 38.2%, in the second in 61.1%, and in the third in 0.7% of the cases. The femoral aperture site did not affect the cartilage status.

A statistically significant correlation was found between both worsening of the cartilage status in the patellofemoral compartment (PFC) (*R*_*s*_ = 0.28, *p* = 0.0012) and medial tibial cartilage (*R*_*s*_ = 0.24, *p* = 0.003) in relation to the position of tibial bone tunnel in the frontal plane.

Cartilage degeneration on the medial side of the knee, either femoral or tibial, was correlated with the tibial aperture site of the tibial tunnel in the lateral view (*R*_*s*_ = 0.28, *p* < 0.001).

Worsening of the cartilage status on the lateral compartment of the knee, femoral or tibial, was found to be inversely correlated with the femoral roof angle (*R*_*s*_ = −0.1985, *p* = 0.02).

Meniscal tears, either at the medial or lateral site or both, were found in 93 patients (60%) during primary ACL-R and increased to 132 patients (86%) during revision ACL-R (Table 4). All these patients received partial meniscal resection during primary ACL-R, however, no significant impact on cartilage degeneration was found, in either the medial nor lateral compartment.

**Table 4:** Medial and lateral meniscal tear at the primary and revision ACL-R

	Medial meniscus	Lateral meniscus	Medial and lateral meniscus
Primary ACL-R	62 (40.3%)	55 (35.7%)	24 (15.6%)
Revision ACL-R	36 (23.4%)	36 (23.4%)	33 (21.4%)

The traumatic and nontraumatic groups comprised 89 and 58 patients, respectively. The remaining seven patients complained about chronic knee instability and were excluded from analysis. Analysis of data between traumatic and nontraumatic failure after primary ACL-R showed that only the aperture of the tibial tunnel in the coronal plane outside the recommended reference of 42–44% had a significant impact between the two groups (Table 5). A difference in cartilage status was only found at the lateral femoral condyle between the two groups, with an increase in degeneration in the traumatic group (*p* = 0.004).

**Table 5:** Factor analysis between traumatic and nontraumatic ACL failure

Parameter	*p*-Value
Female	0.162
Transtibial drilling	0.074
Smaller graft diameter	0.079
Greater angulation of the femoral tunnel in the AP view	0.97
Greater FTA	0.08
Greater FcA	0.066
Intraarticular aperture of femoral tunnel outside the reference of 19–25%	0.145
Intraarticular aperture of tibial tunnel outside the reference of 42–44%	0.018
Tibial slope of more than 12°	0.837

## Discussion

This study showed a significant increase in cartilage degeneration at a mean of 46 months after ACL-R. A significant increase in cartilage degeneration was found in all three knee compartments. While 62.3% of the patients did not show any cartilage degeneration at the time of primary ACL-R, this number decreased to 33.1% in the current young study population after 4 years of follow-up. Progression of cartilage degeneration was predominantly found at the medial femorotibial compartment, followed by the lateral and the patellofemoral compartment (Fig. 1). One may expect accelerated cartilage degeneration at the site where bone bruises occur during ACL injury, as seen on MRI, typically at the posterolateral tibia plateau and the lateral femoral condyle [21]. The distribution of bone bruises was analyzed in a review, showing 35.5% on the lateral femoral condyle, followed by 41% on the lateral tibial plateau, 15% on the medial tibial plateau, and 8% on the medial femoral condyle [8]. However, the accelerated cartilage degeneration at the medial femorotibial compartment may rely on the adduction moment causing an increase in loading. Kinematic studies have previously demonstrated that ACL-R does not restore normal knee function [35]. While anterior tibial translation was similar to the healthy contralateral side, more external rotation and adduction was found, increasing the anterior contact point on the lateral tibial plateau [34]. Altered knee kinematics may have an impact on the magnitude of stress applied to the cartilage. Change in kinematics is caused by the differences in bony morphology, ACL injury, and reconstruction [17, 18]. Anatomic graft geometry showed impacts on kinematics, such as tibial contact location and sliding velocity, on both the medial and lateral tibial plateau [36].

Anatomical variation of the insertion site and accuracy of tunnel placement are challenges in ACL reconstruction, and some kind of knee instability or even tightness after reconstruction may occur as a result [6, 30]. Pathological ACL graft tension can be expected after incorrect tunnel placement and may accelerate cartilage degeneration. However, identification of the exact insertion site on both the femur and tibia is very demanding due to the significant variation of the natural ACL insertion site in terms of dimension, geometry, and fiber bundle orientation [30].

Position of the intraarticular aperture site of the current study was 26% of the entire anteroposterior dimension of the lateral femoral condyle measured in the lateral view. These results are comparable to the intraarticular aperture site analyses in a literature review of 11 studies [40]. Correct femoral tunnel placement in the anteroposterior dimension according to the lateral view in the present study may explain why no impact on cartilage degeneration was observed. However, the lateral view does not allow correct aperture site assessment perpendicular to the anteroposterior measurements. More exact measurements will require computer tomography.

In this study, tibial tunnel position showed more impact on cartilage degeneration progression than femoral tunnel position. The intraarticular aperture at the tibial site was 42.1 ± 3.2% in the coronal and 40.4 ± 5.2% in the sagittal plane, considering the entire anteroposterior and mediolateral distance as reference (Table 3). A tibial insertion site outside the range of 42–44% significantly increased the risk for primary ACL-R failure according to the current study (Table 4). There was a tendency for more medial tibial tunnel placement in the current study than the natural insertion site of the ACL. A more medially placed tibial tunnel may increase rotational stability of the knee, however, the impact on femorotibial contact and force might be of concern. The tibial tunnel was placed 40.4% in the sagittal plane, which is slightly anterior to the natural ACL insertion site of 42%, but inside the range of 38.5–45.5% (5th to 95th percentile) according to the review of 1393 articles [26]. A high percentage (45%) of partially anatomical tibial tunnel placement has been reported [1]. Less than 50% overlap between the natural ACL footprint and the tibial aperture site of the tunnel has been reported in 22 of 40 patients during ACL-R [27]. The significant impact of tibial tunnel placement on knee anteroposterior and rotational stability was shown in a cadaveric study [5]. The present results are in contrast to other studies reporting a more posterior placement of the tibial tunnel in the sagittal plane in order to achieve proper femoral tunnel placement when using the transtibial technique [16]. The more anterior placement of the tibial tunnel may cause notch impingement, especially when the femoral tunnel is placed more superiorly, which in general occurs with the transtibial drilling technique. The most pronounced effect on notch impingement was reported when tibial ACL insertion was shifted anterolaterally by 3 mm, which caused an increase in impingement force of 242.9% [25]. Graft impingement may prevent full extension of the knee, increase both femorotibial and patellofemoral contact, and may accelerate cartilage degeneration [31]. Tibial graft fixation in 5° of knee hyperextension does show a significant impact on knee extension during the first 12 weeks [41]. A weak, yet significant correlation was found between the roof angle, which averaged 33.7 ± 4.6°, and cartilage degeneration in the present study. The roof angle seems to range between 23° and 60° [3]. A decrease in the roof angle increases the risk of graft impingement, and notchplasty might be required during ACL-R. The roof angle should be measured in the lateral view prior to surgery to respect the individual shape of the notch during ACL-R.

Beside tunnel placement, the impact of partial meniscal resection should be emphasized. Meniscal lesions increased from 60% to 86% in all patients between primary and revision ACL-R. Partial meniscal resection during ACL-R causes more knee swelling and inferior outcomes according to International Knee Documentation Committee (IKDC) and Lysholm scoring [13, 39]. More radiographic abnormalities were also reported [39]. Partial medial and lateral meniscal resection during ACL-R causes an increase in radiographic signs of osteoarthritis at the medial (OR, 2.1) and lateral (OR, 2.97) compartment at a minimum follow-up of 2 years [11]. However, no correlation between meniscal lesion and progression of cartilage degeneration has been demonstrated, although these findings presented at an early stage after 15 months of follow-up [4].

To analyze the potential impact of bone tunnel placement, tibial slope, roof angle, and history of failure, the study cohort was divided into patient groups with and without a history of adequate trauma that caused revision ACL-R. Tibial tunnel placement outside the range of 42–44% in the coronal plane showed an impact. None of the other factors had any impact on the cause of failure. More frequently, tunnel enlargement was noticed at the tibial aperture side, presumable causing more stress on the tibial bone [38]. Traumatic and nontraumatic causes for ACL-R failure were recently studied [10]. Tibial tunnel placement did not show any impact in their study and the authors concluded that other factors, such as insufficiency in muscle function, might be more relevant for failure.

This present study is not without limitations. First, the study was performed retrospectively. However, cartilage staging was performed by the same group of surgeons at primary and revision ACL-R and high consistency in cartilage assessment can be presumed. Second, a three-dimensional analysis of the bone tunnels was impossible due to the lack of routine computed tomography (CT) scans or MRI images.

## Conclusion

The present study has demonstrated accelerated cartilage degeneration in all three compartments after failed ACL-R, irrespective of a second adequate or inadequate trauma. Tunnel placement had significant impact on cartilage degeneration. Surgeons should be aware of the importance of tunnel placement during revision ACL-R, to reduce the risk of a potential revision surgery.

## Data Availability

All data are available an anonymized according to the data protection guidelines.
